# Wound-induced polyploidization is driven by Myc and supports tissue repair in the presence of DNA damage

**DOI:** 10.1242/dev.173005

**Published:** 2019-08-02

**Authors:** Janelle Grendler, Sara Lowgren, Monique Mills, Vicki P. Losick

**Affiliations:** Kathryn W. Davis Center for Regenerative Biology and Medicine, MDI Biological Laboratory, 159 Old Bar Harbor Rd, Bar Harbor, ME 04609, USA

**Keywords:** DNA damage, Endoreplication, Myc, Polyploidy, Regeneration, Yorkie

## Abstract

Tissue repair usually requires either polyploid cell growth or cell division, but the molecular mechanism promoting polyploidy and limiting cell division remains poorly understood. Here, we find that injury to the adult *Drosophila* epithelium causes cells to enter the endocycle through the activation of Yorkie-dependent genes (*Myc* and *E2f1*). Myc is even sufficient to induce the endocycle in the uninjured post-mitotic epithelium. As result, epithelial cells enter S phase but mitosis is blocked by inhibition of mitotic gene expression. The mitotic cell cycle program can be activated by simultaneously expressing the Cdc25-like phosphatase String (*stg*), while genetically depleting APC/C E3 ligase fizzy-related (*fzr*)*.* However, forcing cells to undergo mitosis is detrimental to wound repair as the adult fly epithelium accumulates DNA damage, and mitotic errors ensue when cells are forced to proliferate. In conclusion, we find that wound-induced polyploidization enables tissue repair when cell division is not a viable option.

## Abstract (precis)

**Summary:** Wound-induced polyploidization in the *Drosophila* epithelium is dependent on Myc and enables tissue repair in the presence of DNA damage when cell division is not a viable option.

## INTRODUCTION

Regeneration is limited for many organs due to the lack of a resident stem cell or progenitor cell population. As result, when injury or damage occur, organ failure may be delayed by the growth of cells through polyploidy (Lazzeri et al., 2019). Polyploidy is the more than doubling of the genome of a cell and frequently arises during organogenesis, tissue repair and disease (Gjelsvik et al., 2019). Polyploid cells are generated by both cell cycle-dependent and -independent mechanisms, including endoreplication and cell fusion. Endoreplication is an incomplete cell cycle that can generate either a binucleated or mononucleated polyploid cell via endomitosis or the endocycle. Cell fusion also allows the formation of multinucleated, polyploid cells independently of the cell cycle. By becoming polyploidy, cells are able to grow by orders of magnitude, as cell size is known to be proportional to DNA content (Frawley and Orr-Weaver, 2015).

Polyploidy has been found to either act as a barrier or as a driver of tissue repair and regeneration (Gjelsvik et al., 2019). In the heart, polyploid cardiomyocytes have been shown in both the mouse and zebrafish to impair regenerative capacity (Patterson et al., 2017; González-Rosa et al., 2018). In the zebrafish epicardium, however, polyploid cells are generated during wound healing and act to promote regeneration by enhancing the rate of wound closure (Cao et al., 2017). Acute injury to the mouse kidney also results in the generation of polyploid cells in the tubule epithelium through endoreplication, which acts to compensate for cell loss and restore organ function (Lazzeri et al., 2018). In the mammalian liver, hepatocytes both divide and grow as polyploid cells, although endoreplication is dispensable for liver regeneration (Pandit et al., 2012; Zhang, et al., 2018; Wilkinson et al., 2019).

As in vertebrates, polyploidy frequently arises in the fruit fly in response to tissue damage (Gjelsvik et al., 2019; Ovrebo and Edgar, 2018). In *Drosophila*, the abdominal epithelium, hindgut pylorus, intestine, as well as follicular epithelium, generate polyploid cells, albeit using different mechanisms, to promote tissue repair (Losick et al., 2013, 2016; Cohen et al., 2018; Xiang et al., 2017; Tamori and Deng, 2013). Polyploid growth has the ability, at least in the fly epithelium and hindgut, to restore tissue mass with fewer, yet larger cells (Losick et al., 2013, 2016; Cohen et al., 2018). One key advantage of polyploidy is that wound repair can occur in the absence of cell division, but the change in cell size can permanently alter the tissue organization. Therefore, many studies have focused on activating cell proliferation to promote tissue regeneration instead.

One mechanism for switching modes of tissue growth between cell proliferation and polyploidization is dependent on regulation of the cell cycle. It is feasible to switch from a mitotic cell cycle to an endocycle, or vice versa, by regulating mitotic gene expression. Endoreplication requires the downregulation of mitotic cyclins, which are targeted by Fizzy-related (Fzr), an E3 ubiquitin ligase in *Drosophila* (Sigrist and Lehner, 1997; Zielke et al., 2008; Maqbool et al., 2010). Genetic loss of key M-phase regulators, such as cyclin dependent kinase 1 (Cdk1), in either *Drosophila* or the mouse liver inhibited cell division causing cells to default into an endocycle (Hayashi, 1996; Weigmann et al., 1997; Diril et al., 2012). Remarkably polyploidy was sufficient to maintain tissue growth and organ size (Weigmann et al., 1997; Diril et al., 2012). Genetic activation of mitotic regulators has also been demonstrated to effectively switch cells from polyploidization to proliferation in both *Drosophila* and mammalians cells (Djabrayan et al., 2014; Cohen et al., 2018; Mohamed et al., 2018). However, the immediate and long-term effects of switching modes of tissue growth for tissue repair and regeneration remaining largely unknown. Recent studies in the *Drosophila* hindgut and mammalian liver have shown that cells can be reprogrammed to a mitotic cell cycle and still effectively regenerate, but the switch to cell proliferation has the long-term effect of sensitizing the tissue to oncogenic growth (Cohen et al., 2018; Zhang et al., 2018; Wilkinson et al., 2019).

**Fig. 1.**
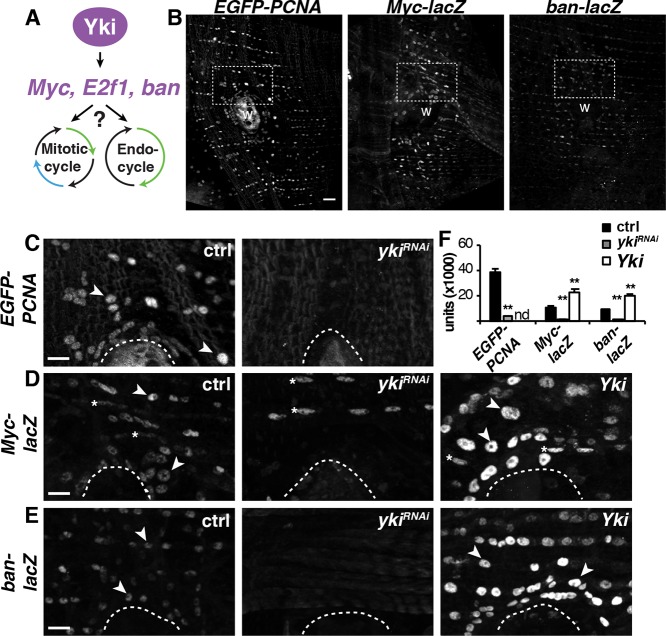
**Yki-dependent cell cycle and growth genes are induced during WIP.** (A) Model illustrating the regulation of both mitosis and the endocycle by Yki*.* (B) Immunofluorescent images of known Yki targets are induced around the wound site: EGFP-PCNA (E2f1 reporter) and *Myc-lacZ* at 2 dpi, and *ban-lacZ* at 1 dpi. Scale bar: 50 µm. Wound scab (W). (C-E) Higher magnification of the boxed areas in B for the control (*w^[1118]^*) and epithelial-specific knockdown (*yki^RNAi^*) or overexpression (*Yki*) using the Gal4/ UAS system. Arrowheads indicate examples of epithelial nuclei expressing the reporter gene of interest; asterisks indicate muscle nuclei expressing *Myc-lacZ* in D. Wound scab is indicated by the dashed line. Scale bars: 10 µm. (F) Quantification of Yki-dependent reporter expression in epithelial nuclei around the wound site for EGFP-PCNA, NP2108-Gal4 [ctrl (*n*=151); *yki^RNAi^* (*n*=150)]; *Myc-lacZ*, epi-Gal4 [ctrl (*n*=100), *yki^RNAi^* (*n*=100) and *Yki* (*n*=100)]; and *ban-lacZ*, epi-Ga14 [ctrl (*n*=150), *yki^RNAi^* (*n*=100) and *Yki* (*n*=150)]. **P<0.01. (Also see Table S1.)

Another unanswered question is how are these distinct modes of tissue growth induced during tissue repair and regeneration? In response to injury or damage, polyploid cell growth has been found to be regulated by several conserved signaling pathways, including Hippo, JNK, InR and EGFR in *Drosophila* (Losick et al., 2013, 2016; Tamori and Deng, 2013; Xiang et al., 2017). In zebrafish epicardium, transient polyploid cell growth during heart regeneration has been shown to be enhanced by mechanical tension (Cao et al., 2017). However, these signaling pathways are also key drivers of cell proliferation. Therefore, it remains unknown how similar signals can result in distinct tissue repair outcomes, i.e. cell proliferation versus polyploidization (Fig. 1A).

Here, we show in the adult fruit fly model of wound healing that the lack of epithelial cell division is based on the same principle as that seen during developmentally programmed polyploidy. The epithelial cell mitotic machinery of the adult fruit fly is permanently turned off, in part by constitutive expression Fzr (Sigrist and Lehner, 1997; Zielke et al., 2008). As a result, injury activates transcriptional activator Yorkie (Yki), leading to induction of cell cycle regulatory genes, including *Myc* and *E2f1*. Expression of either *Myc*, *E2f1* or an E2f1 target, *CycE*, is necessary and sufficient for the fly epithelial cells to enter S phase and initiate the endocycle post-injury. We further show that the mitotic cell cycle can be genetically activated during wound repair but doing so results in mitotic errors and defects in re-epithelialization. The adult *Drosophila* epithelium accumulates low levels of DNA damage, which can be enhanced by exposure to UV irradiation. As a result, wound-induced polyploidization (WIP) enables tissue repair in presence of DNA damage. Therefore, polyploid growth is advantageous for tissue repair by permitting wound healing when cell division would cause harm to tissue homeostasis.

## RESULTS

### Yki-dependent targets are required and sufficient for endoreplication during WIP

Our previous studies found that Yki regulates WIP by controlling the endocycle post-injury in the adult *Drosophila* epithelium; however, the required Yki transcriptional targets remained unknown (Losick et al., 2013, 2016). Yki is the co-transcriptional activator of the Hippo pathway and is known to induce expression of several cell cycle and growth genes, including *Myc*, *E2f1*, *CycE* and the microRNA *bantam* (*ban*) (Nicolay et al., 2011; Oh et al., 2013; Slattery et al., 2013; Ikmi et al., 2014). All of these genes have been linked to controlling endoreplication in tissue development by controlling G/S transition (Lilly and Spradling, 1996; Pierce et al., 2004; Zielke et al., 2011; Jiang et al., 2014). Therefore, we hypothesized that Yki-dependent induction of a similar gene set may also be driving the adult epithelial cells to enter the endocycle post-injury (Fig. 1A). To investigate this hypothesis, we monitored the expression of these genes using the reporters: EGFP-PCNA fusion protein, *Myc-lacZ* and *ban-lacZ* (Deneke et al., 2016; Neto-Silva et al., 2010; Brennecke et al., 2003). Reporter expression in the control (ctrl) fruit flies was induced at 2 days post-injury (dpi) in epithelial cells surrounding the wound site (Fig. 1B). Yki expression was then either knocked down (*yki^RNAi^*) or overexpressed (*Yki*) in the adult fly epithelium using the previously characterized epithelial specific Gal4 driver, epi-Gal4 (Losick et al., 2016, 2013). Indeed, we observed that all wound-induced expression was dependent on Yki (Fig. 1C-E). Epithelial*-*specific *yki* knockdown reduced EGFP-PCNA protein expression as well as *Myc-lacZ* and *ban-lacZ* reporter gene expression, whereas *Yki* overexpression significantly enhanced *Myc-lacZ* and *ban-lacZ* reporter gene expression around the wound site (Fig. 1F). Because EGFP-PCNA and epi-Gal are inserted at the same attP2 site in the *Drosophila* genome, *yki* expression was regulated with another epithelial driver NP2108-Gal4 (Scherfer et al., 2013). NP2108-Gal4 is not specific to the adult epithelium; as a result, Yki overexpression was lethal and could not be determined (Fig. 1F).

**Fig. 2.**
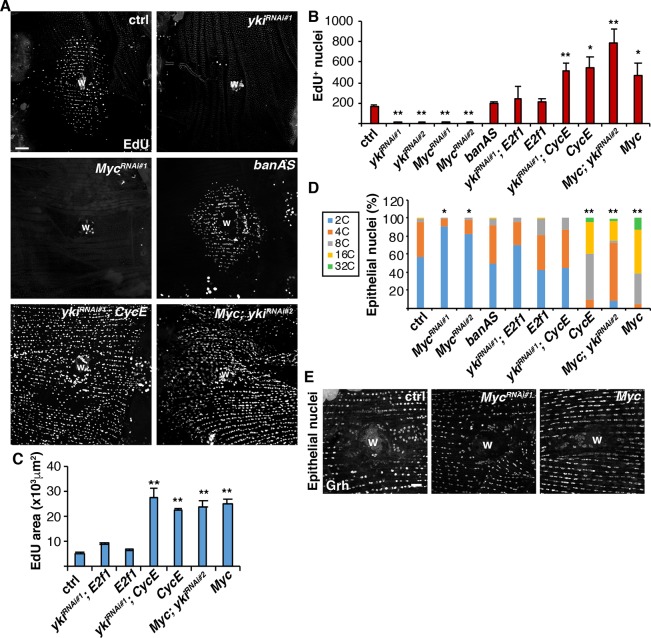
***Myc*, *E2f1* and *CycE* are required and sufficient for endoreplication in WIP.** (A) Immunofluorescent images of EdU labeling in fly abdomens expressing the denoted gene using epi-Gal4/UAS system at 2 dpi. Wound scab (W). Scale bar: 50 µm. (B) Average number of EdU^+^ epithelial nuclei per fly at 2 dpi: control (*n*=37), *yki^RNAi#1^* (*n*=13), *yki^RNAi#2^* (*n*=5), *Myc^RNAi#1^* (*n*=10), *Myc^RNAi#2^* (*n*=7), *banAS* (*n*=9), *yki^RNAi#1^, E2f1* (*n*=10), *E2f1* (*n*=6), *yki^RNAi#1^, CycE* (*n*=7), CycE (*n*=7), *Myc; yki^RNAi#2^* (*n*=9) and *Myc* (*n*=8). (C) Average area labeled with EdU^+^ nuclei: control (*n*=27), *yki^RNAi#1^; E2f1* (*n*=9), *E2f1* (*n*=6), *yki^RNAi#1^; CycE* (*n*=7), CycE (*n*=7), *Myc; yki^RNAi#2^* (*n*=9) and *Myc* (*n*=8). (D) Epithelial nuclear ploidy (%) at 3 dpi: control (*n*=4), *Myc^RNAi#1^* (*n*=6), *Myc^RNAi#2^* (*n*=5), *banAS* (*n*=4), *yki^RNAi#1^; E2f1* (*n*=4), *E2f1* (*n*=4), *yki^RNAi#1^, CycE* (*n*=3), CycE (*n*=3), *Myc; yki^RNAi#2^* (*n*=5) and *Myc* (*n*=3). *P<0.05; **P<0.01. (E) Epithelial nuclear size (labeled using anti-Grh) is altered by *Myc* expression at 3 dpi. Scale bar: 20 µm. (Also see Table S2.)

Next, we tested whether these Yki-dependent targets are required for entry into the endocycle during WIP by first quantifying the number of EdU^+^ epithelial nuclei as an indicator of S-phase entry at 2 dpi (Fig. 2A). Epithelial specific knockdown of *Myc* significantly reduced *Myc-lacZ* expression by 1.5 fold (Table S8, *t*-test, *P*<0.05) and also inhibited entry into S phase, similar to the knockdown of *E2f1* or *CycE*, which we found previously to be required for endoreplication (Fig. S1A,B and Fig. 2A,B) (Losick et al., 2013). However, inhibition of *bantam* (*ban*) by expression of an antisense inhibitor (*banAS*) failed to inhibit S-phase entry (Fig. 2A,B). We confirmed that epithelial expression of the *banAS* inhibitor efficiently blocked *ban* expression post-injury (Fig. S1C,D and Table S8). In addition, *Myc-lacZ* was still expressed when *ban* was inhibited (Fig. S2 and Table S9), indicating that *E2f1* and *Myc* are at least two of the key Yki targets required for entry into the endocycle during WIP.

*ban* and *CycE* were previously identified as essential regulators of tissue growth and are sufficient even in the absence of *yki* to promote cell proliferation in *Drosophila* imaginal discs (Thompson and Cohen, 2006; Meserve and Duronio, 2015; Shu and Deng, 2017)*.* To determine whether Yki-dependent genes are sufficient to induce endoreplication during WIP, we simultaneously suppressed *yki* by RNAi in the adult fruit fly epithelium while overexpressing either *E2f1*, *CycE* or *Myc.* We validated the genomic insertion of the UAS-*yki^RNAi^* animals and confirmed efficient *yki* knockdown in the adult fruit fly epithelium for the conditions tested (Fig. S3 and Table S10). Remarkably, the overexpression of each gene was sufficient to rescue the *yki* knockdown, restoring the capacity of epithelial cells to enter the S phase at 2 dpi (Fig. 2A,B).

Next, we measured ploidy to determine whether the enhanced epithelial S phase entry was occurring via endoreplication. We used our previously developed semi-automated method to quantify the distribution and ploidy of most nuclei throughout the repaired abdominal epithelium (Losick et al., 2016). As expected at 3 dpi, the epithelium surrounding the wound was composed of 44% polyploid nuclei with DNA content more than 3C (Fig. 2D). Overexpression of *CycE* in the adult fly epithelium resulted in 100% polyploid epithelial nuclei at 3 dpi (Fig. 2D), consistent with EdU analysis (Fig. 2B). Overexpression of *Myc* also resulted in 100% polyploid epithelial nuclei around the wound site and the percentage was not significantly reduced by knockdown of *yki (Myc; yki ^RNAi^*), unlike *CycE* and *E2f1*, which were halved (Fig. 2D). We confirmed that *Myc* knockdown resulted in a significant block in endoreplication as only 9% of epithelial nuclei were polyploid in the *Myc^RNAi#1^* condition and 17% in the *Myc^RNA#2^* condition. Epithelial nuclear size was also visibly affected by *Myc* expression with either reduced or enlarged nuclei present as detected by staining for the epithelial-specific transcription factor Grainyhead (Grh) (Fig. 2E).

### Myc is sufficient to drive endoreplication even in uninjured, post-mitotic epithelial cells

**Fig. 3.**
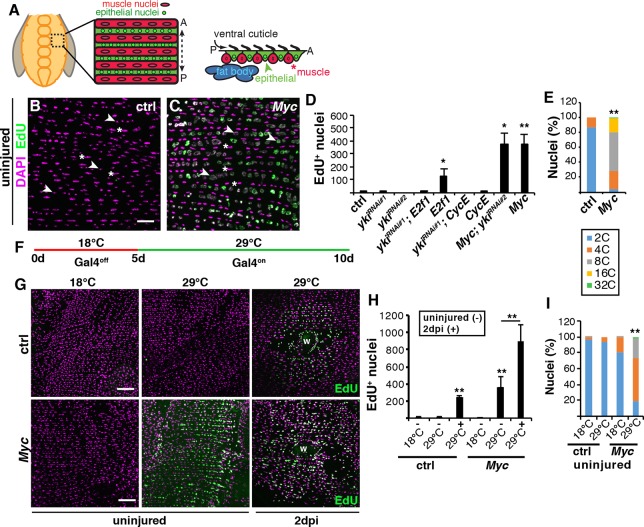
**Myc is a potent inducer of polyploidization in post-mitotic epithelial cells.** (A) Illustration of the adult *Drosophila* abdominal tissue organization. A continuous epithelium underlies the cuticle exoskeletal with overlaying lateral muscle fibers that organize the epithelial nuclei into rows. (B,C) EdU labeling in uninjured adult epithelium. EdU-labeled nuclei (green) and DAPI (magenta). Arrowheads and asterisks indicate epithelial and muscle nuclei, respectively. Scale bar: 50 µm. (D) Quantification of the average number of EdU^+^ nuclei per fly for control (*n*=5), *yki^RNAi#1^* (*n*=6), *yki^RNAi#2^* (*n*=3), *yki^RNAi#1^; E2f1* (*n*=4), *E2f1* (*n*=3), *yki^RNAi#1^; CycE* (*n*=3), CycE (*n*=5), *Myc; yki^RNAi#2^* (*n*=5) and *Myc* (*n*=3). Genes expressed using epi-Gal/UAS and examined after 5 days on an EdU-rich diet. (E) Epithelial nuclear ploidy (%) in control and *Myc* uninjured epithelium (*n*=3). (F) Schematic of conditional Gal4 regulation, where epi-Gal4 is off at a restrictive temperature (18°C) and on at a permissive temperature (29°C). (G) EdU staining (green) at 18°C or 29°C in uninjured (−) epithelium or in epithelium at 2 dpi (+). DAPI (magenta). Wound scab, W. Scale bars: 50 µm. (H) Quantification of S-phase entry for control [18°C (*n*=3), −29°C (*n*=4), +29°C (*n*= 3)] and *Myc* [−18°C (*n*=4), −29°C (*n*=4), +29°C (*n*=3)]. (I) Epithelial nuclear ploidy (%) in uninjured epithelium for control [18°C (*n*=5) and 29°C (*n*=4)] and *Myc* [18°C (*n*=3), 29°C (*n*=5)]. *P<0.05; **P<0.01. (Also see Table S3.)

We questioned whether overexpression of *CycE* or *Myc* was sufficient to induce polyploidy without injury given that the overexpression resulted in 100% polyploid epithelial cells surrounding the wound site (Fig. 2D). Indeed, the uninjured adult epithelial nuclei robustly labeled with EdU and visibly enlarged in size when *Myc* was overexpressed alone or in combination with *yki^RNAi^* (Fig. 3A-D). The only other genetic condition that slightly enhanced EdU incorporation in the uninjured epithelium was the expression of *E2f1* alone (Fig. 3D). We confirmed that EdU labeling was consistent with an increase in polyploidization, as *Myc* overexpression resulted in 96% of epithelial nuclei being polyploid (3C-32C) when compared with the control, epi-Gal4 alone, which was only 13% polyploid (Fig. 3E).

We also observed that epithelial cell size was significantly increased when *Myc* was overexpressed with both enlarged mono- and multinucleated epithelial cells visible (Fig. S4A-D and Table S11). This would be expected, as cell size is known to scale with DNA content (Frawley and Orr-Weaver, 2015). However, the continuous overexpression of *Myc* in the adult fruit fly epithelium led to abnormalities in the epithelial organization, including a reduction in total cell number (Fig. S4C and Table S11), which could be indicative of cell death. *Myc* overexpression has been shown to be an inducer of apoptosis (Bellosta and Gallant, 2010). *Myc* was found to induce the apoptotic gene *hid* and we monitored *hid-GFP* expression in the epithelium (de la Cova et al., 2004). However, we found that *hid-GFP* expression was not altered by *Myc* in this tissue (Fig. S4E and Table S11). In addition, we quantified the total number of epithelial nuclei and found that epithelial nuclear number did not significantly change, despite the reduction in cell number (Fig. S4C and Table S11). Taken together, we have found that *Myc* is required and sufficient to induce endoreplication by activating entry into S phase and not through the induction of cell death.

Next, *Myc* expression was conditionally regulated to test whether *Myc*-dependent endoreplication was sufficient to induce polyploidy in the post-mitotic epithelium and not due to the continuous overexpression of *Myc* during development. Epi-Gal4 expression was controlled using a temperature-sensitive Gal80 inhibitor (Gal80^ts^) (McGuire et al., 2004). The epi-Gal4 driver is only activated and *Myc* expression induced when adult fruit flies were shifted to the permissive temperature (29°C) at 5 days of age (Fig. 3F). Under these regulated conditions, the uninjured adult epithelial nuclei incorporated EdU after shift to 29°C (Fig. 3G-I). At the restrictive temperature (18°C), epithelial cells failed to enter S phase and did not endocycle, demonstrating that *Myc* is a potent inducer of endoreplication in post-mitotic epithelium. We still observed a fourfold increase in the percentage of multinucleated epithelial cells, from 3% in control to 13% in the Gal80^ts^; epi-Gal4, UAS-Myc *Drosophila* strain at 18°C (Fig. S4F and Table S11), but this alternation in epithelial organization was not sufficient to drive endoreplication (Fig. 3H,I). There appears to be some variability in epithelial organization in *Drosophila* strains, as it has previously been reported that ∼60% of the adult fly epithelium becomes multinucleated by 3 days of age (Scherfer et al., 2013). However, we only observe binucleation in 6% of fly epithelial cells in 3-day-old flies with no indication that the epithelium deteriorates until 20 days of age (Fig. S4A,D,F and Table S11) (A. Dehn and V.P.L., unpublished).

### The mitotic cell cycle can be activated by genetically modulating the cell cycle machinery

**Fig. 4.**
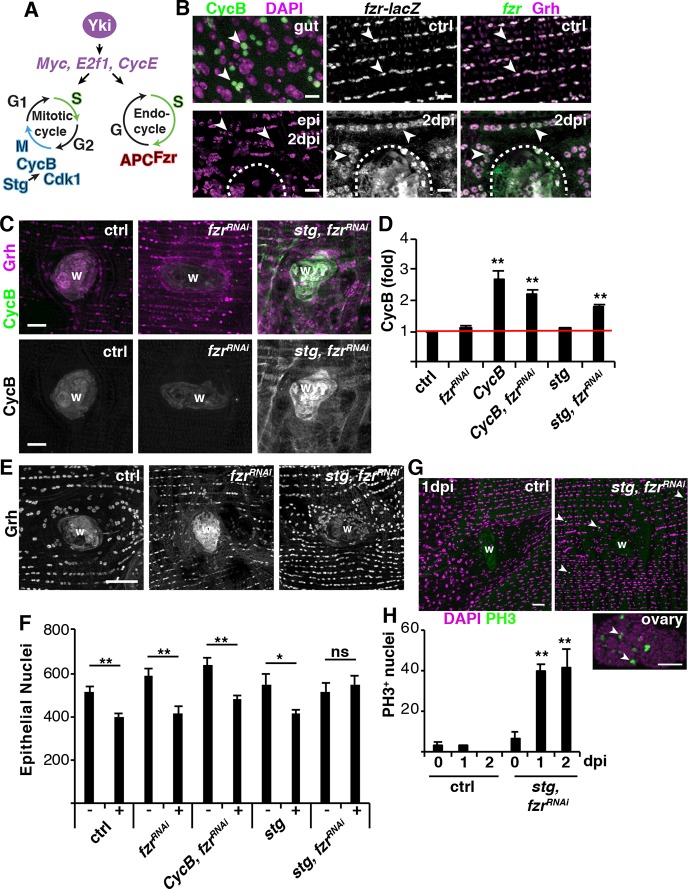
**The mitotic cell cycle can be activated post-injury.** (A) Model illustrating key proteins required for either the mitotic cycle or the endocycle*.* (B) Immunofluorescent images of CycB-GFP (green) in proliferating fly intestinal stem cells and adult epithelium at 2 dpi as well as *fzr-lacZ* reporter (green) in abdominal epithelium (Grh, magenta). Wound scab is indicated by a dashed line. Arrowheads indicate examples of either gut or abdominal cells. Scale bars: 10 µm. (C) Immunofluorescent images of CycB staining in control, *fzr^RNAi^* and *stg*, *fzr^RNAi^* at 3 dpi. Anti-CycB (green) and anti-Grh (magenta). Wound scab (W). Scale bars: 20 µm. (D) Quantification of the fold induction of CycB protein intensity [control (*n*=3), *fzr^RNAi^* (*n*=7), *CycB* (*n*=8), *fzr^RNAi^, CycB* (*n*=6), *stg* (*n*=6) and *stg, fzr^RNAi^* (*n*=6)]. (E) Enhanced epithelial nuclear number (Grh) when cells are switched to the mitotic cycle with *stg, fzr^RNAi^* expression at 3 dpi. Scale bar: 50µm. (F) Quantification of the epithelial nuclear number in both uninjured (−) and 3 dpi (+) abdomens: control (−, *n*=11; +, *n*=13), *fzr^RNAi^* (−, *n*=7; +, *n*=8), *fzr^RNAi^, CycB* (−, *n*=10; +, *n*=13), *stg* (−, *n*=6; +, *n*=8) and *stg, fzr^RNAi^* (−, *n*=8; +, *n*=9). (G) Immunofluorescent images of PH3 (green) staining in control and *stg, fzr^RNAi^* abdomens at 1 dpi, as well as in the fly ovary, where cells are known to proliferate. DAPI (magenta). Arrowheads indicate examples of PH3^+^ cells. Scale bars: 20 µm. (H) Quantification of the average number of PH3^+^ epithelial nuclei per fly: control [0 dpi (*n*=3), 1 dpi (*n*=4) and 2 dpi (*n*=5)] and *stg, fzr^RNAi^* [0 dpi (*n*=5), 1 dpi (*n*=3) and 2 dpi (*n*=4)]. *P<0.05; **P<0.01; ns, not significant. (Also see Table S4.)

Mitotic cyclin suppression is a prerequisite for endoreplication initiation, and developmental studies in *Drosophila* have shown that Fizzy-related ubiquitin ligase (Fzr) is required for the endocycle and acts by targeting cyclins A, B and B3 for proteolytic degradation (Fig. 4A) (Sigrist and Lehner, 1997; Zielke et al., 2008). Consistent with these earlier observations, we found that Fzr is constitutively expressed, while CycA and CycB expression are repressed in the post-mitotic fly epithelium even after injury (Fig. 4B and Fig. S5E). The *fzr-lacZ* reporter colocalizes with the epithelial-specific marker Grh as well as the epi-Gal4, UAS-nlsRFP marker, and is not expressed in the overlaying lateral muscle fiber nuclei (Fig. 4B and Fig. S5A,B). Therefore, we hypothesized that Yki induces entry into S phase through expression of *Myc* and *E2f1*, while the constitutive expression of Fzr simultaneously reduces the levels of mitotic cyclin proteins to promote endoreplication, instead of proliferation (Fig. 4A).

Next, we determined whether the mitotic cell cycle can be activated by genetically modulating the levels of the cell cycle machinery. Epithelial cells enter the cell cycle between 24 and 48 h post injury, leaving a large window to capture a mitotic event (Losick et al., 2013). To account for this, we monitored mitotic cell cycle activity by first immunohistochemically staining for CycB protein expression. As a positive control, we observed the accumulation of CycB in actively dividing intestinal stem cells (Fig. 4B). It has recently been found that *fzr* knockdown was sufficient to switch hindgut pyloric cells from injury-induced polyploidy to proliferation (Cohen et al., 2018); however, we found that knocking down *fzr* alone was not sufficient to activate the mitotic cycle as CycB expression was not detected (Fig. 4C,D). We confirmed *fzr* RNAi blocked endoreplication as epithelial nuclear size and ploidy (>3C, 7%±2.7%) were significantly reduced (Fig. 4E; data not shown). In *Drosophila* ovary, simultaneously expressing *String* (*stg*), a *Cdc25* ortholog*,* and genetically removing one copy of *fzr* was sufficient to force follicle cells to go through additional mitotic cell cycles before entering the endocycle (Schaeffer et al., 2004). Indeed, this genetic combination (*stg* overexpression with *fzr* knockdown denoted *stg, fzr^RNAi^*) was sufficient to significantly induce CycB protein expression in the adult fruit fly epithelium post-injury, comparable with overexpression of CycB alone (Fig. 4C,D). However, CycA, another mitotic cyclin targeted by Fzr was not induced by *stg, fzr* RNAi (Fig. S5E).

The induction of CycB protein expression was predominantly adjacent to the wound site (Fig. S5C), even though the epi-Gal4/UAS system robustly drives expression throughout the adult fly ventral epithelium (Fig. S5D) (Losick et al., 2013). Additional regulators may therefore be required to permit CycB protein expression within this wound permissive zone. We found that *stg, fzr^RNAi^* expression also upregulated expression of Geminin protein, a DNA replication initiation inhibitor, adjacent to the wound site (Fig. S5E) (Quinn et al., 2001). Therefore, CycB protein expression may be indirectly activated by induction of Geminin, which would slow the cell cycle.

As a secondary measure of mitotic division, the number of nuclei before and after injury was also quantified. A puncture wound resulted in the loss of 119 epithelial cells on average and because cells grow instead of dividing to heal the wound, epithelial nuclear number is not restored in the control condition post-injury (Fig. 4E,F) (Losick et al., 2013; Losick et al., 2016). Therefore, we would only expect to see a rescue in nuclear number if the mitotic cycle is activated. The only genetic condition that restored epithelial nuclear number post-injury was *stg, fzr^RNAi^*, in which there was no significant difference between the number of nuclei in the uninjured and 3 dpi epithelium (Fig. 4F). We then stained the epithelium with mitotic marker phospho-histone 3 (PH3) to further confirm that *stg, fzr^RNAi^* effectively switched epithelial cells to the mitotic cell cycle. Indeed, we could detect several PH3^+^ epithelial nuclei around the wound site at 1 and 2 dpi, whereas PH3^+^ staining was not observed in the control or uninjured *stg, fzr^RNAi^* abdominal tissue (Fig. 4G,H). Taken together, *stg, fzr^RNAi^* effectively switched the adult abdominal epithelial cells to the mitotic cell cycle post injury. In addition, we found *stg, fzr^RNAi^* did not affect induction of *Myc* expression (Fig. S2H-K), but only altered the area of cells that were competent to enter the cell cycle as PH3^+^ were observed distant from the wound site (Fig. 4G).

### Forced mitotic cell cycle is detrimental to wound repair

**Fig. 5.**
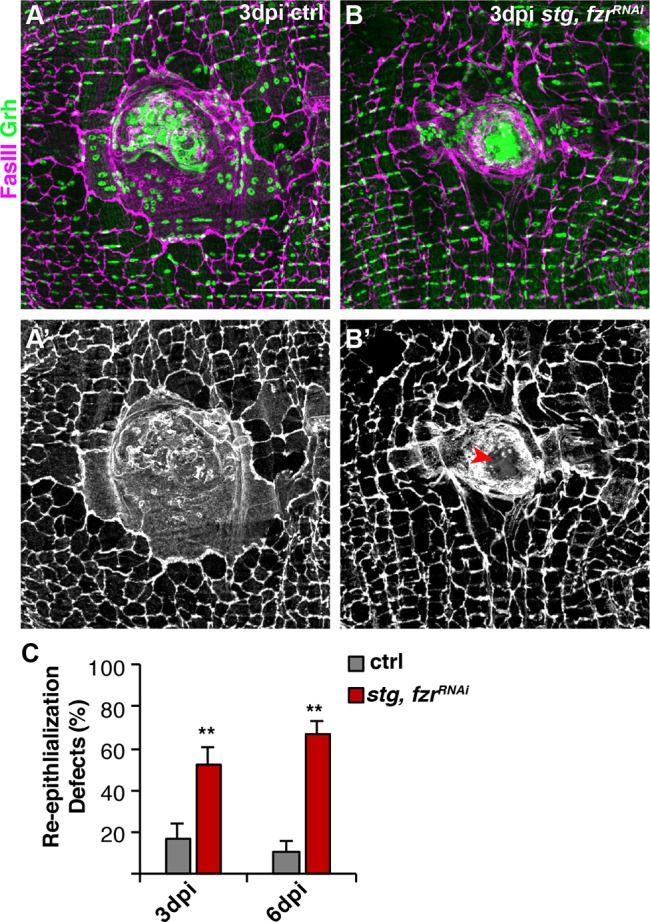
**Activation of the mitotic cycle compromises re-epithelialization.** Re-epithelialization is perturbed when the mitotic cell cycle is induced in adult fly epithelium. Immunofluorescent images of control (A) and *stg, fzr RNAi* (B) at 3 dpi. Epithelial nuclei and septate junctions are stained for Grh (green) and FasIII (magenta), respectively. (A′,B′) FasIII staining alone shows that re-epithelialization is impaired (red arrowhead) in *stg, fzr^RNAi^* flies. Scale bar: 50 µm. (C) Quantification of re-epithelialization defects (%) at 3 dpi (gray) [control (*n*=8), *stg, fzr^RNAi^* (*n*=6)] and 6 dpi (red) [control (*n*=5) and *stg, fzr^RNAi^* (*n*=5)]. **P<0.01. (Also see Table S5.)

Next, we asked whether wound repair was improved by switching to a proliferative response. In the adult fruit fly, WIP occurs by both cell fusion and endoreplication (Losick et al., 2013, 2016). Epithelial cell-to-cell junctions were detected by staining for FasIII, a septate junction protein, to determine whether re-epithelialization was complete. By 3 dpi, WIP repairs the epithelium by forming a giant multinucleated cell that covers the wound scab, restoring a continuous epithelial sheet (Fig. 5A,C). We found that the expression of *stg, fzr^RNAi^*, which activated the mitotic cell cycle, led to significant defects in re-epithelialization at 3 dpi (Fig. 5B,C). Half of the wounds (52%) were not able to form a continuous epithelial sheet over the wound scab with gaps of >10 µm detected (Fig. 5B′, red arrow).

Re-epithelialization was also quantified by visualizing the epithelial membrane by epi-Gal4 expression of UAS-mCD8-ChRFP (Losick et al., 2013). In control, 91% of epithelial wounds close completely by 3 dpi, but inhibiting WIP by blocking endoreplication (*E2f1^RNAi^*) and cell fusion (*RacN17*) simultaneously, as we previously reported, causes 92% of epithelial wounds to remain open (Fig. S6A,B and Table S12). The activation of mitotic cell cycle by expression of *stg, fzr^RNAi^*, however, resulted in a partial epithelial wound closure defect (Fig. S6A,B and Table S12). Defects in re-epithelialization were observed in 62% of *stg, fzr^RNAi^* wounds with noticeable gaps (>10µm) in the epithelial sheet covering the wound scab (Fig. S6A, dashed red outline), consistent with defects observed by with FasIII localization (Fig. 5). In addition, in the 38% of cases where re-epithelialization was observed epithelial membranes appeared to be thinner than in 3 dpi controls (Fig. S6A,C and Table S12). The expression of mCD8-ChRFP over the wound scab was significantly reduced by 2.4-fold, suggesting that epithelial integrity has been compromised (Fig. S6C and Table S12).

### Forced mitotic cell cycle re-entry results in mitotic catastrophe

**Fig. 6.**
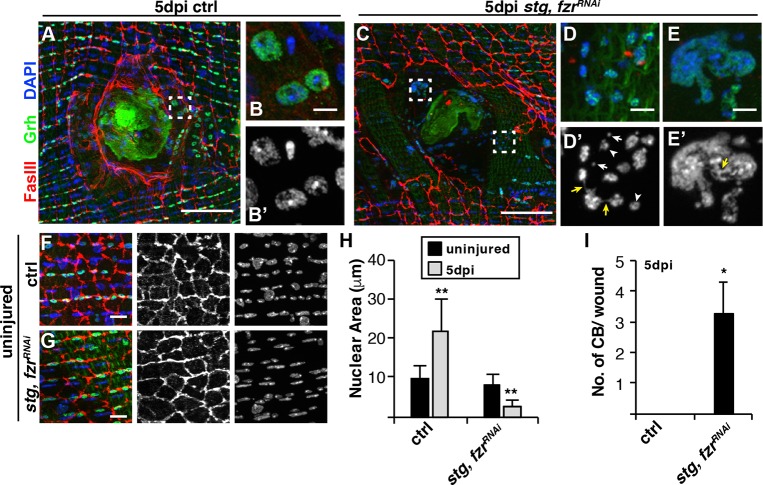
**Mitotic errors occur when the mitotic cell cycle is activated.** (A,C) Immunofluorescent images of control (A) and *stg, fzr^RNAi^* (C) epithelium at 5 dpi. Boxed region in A is shown in B,B′; boxed regions in C are shown in D,D′ (bottom box) and E,E′ (top box). (B,D,E) Merge images; (B′,D′,E′) DAPI alone. Examples of reduced nuclear size (arrowheads), micronuclei (white arrows) and chromatin bridging (yellow arrows). Epithelial nuclei (Grh, green) and septate junctions (FasIII, red), and DAPI (blue). Scale bars: 50 µm in A,C; 5 µm in B,B′,D-E′. (F,G) Immunofluorescent images of uninjured epithelium: control (F) and *stg, fzr^RNAi^* (G). Scale bars: 10 µm. (H) Quantification of the epithelial nuclear area in control and *stg, fzr^RNAi^* in uninjured (−) or at 5 dpi (+), *n*=3. (I) Quantification of the chromatin bridging (CB) events near wound scab (*n*=5). *P<0.05; **P<0.01. (Also see Table S6.)

The rate of tissue repair was shown to be slower when cells relied on cell proliferation instead of polyploidization in mouse liver and zebrafish epicardium (Miyaoka et al., 2012; Cao et al., 2017). Therefore, we allowed wound healing to proceed for an additional 2 days until 5 dpi and investigated whether re-epithelialization had improved. However, at this time point, re-epithelialization remained significantly impaired in the *stg, fzr^RNAi^* flies when compared with 5 dpi control (Figs 5C and 6A,C). Loss of epithelial integrity became more apparent with a discontinuous epithelial sheet covering the wound scab (Fig. 6C).

We also frequently observed abnormal epithelial nuclei near the wound scab, which still labeled with the epithelial marker Grh (Fig. 6C-E). *stg, fzr^RNAi^* epithelial nuclei were reduced in size by ∼3.5-fold compared with uninjured nuclei (Fig. 6B,D,H). Micronuclei were frequently located in the vicinity of epithelial nuclei (Fig. 6D′). The expression of *stg, fzr^RNAi^* did not affect the uninjured epithelium, as epithelial nuclear size and tissue architecture were indistinguishable from controls (Fig. 6F-H). We also observed interconnections between nuclei, either thin chromatin connections or large masses containing multiple nuclei (Fig. 6D,E). These chromatin bridging (CB) events in *stg, fzr^RNAi^* wounds had an average of three CB events near the wound scab, whereas this defect was not detectable in control wounds at 5 dpi (Fig. 6I). Micronuclei and CB are both hallmarks of mitotic catastrophe (Vakifahmetoglu et al., 2008; Surova and Zhivotovsky, 2013), suggesting that re-activation of the mitotic cell cycle may compromise wound repair by leading to mitotic errors that result in further loss of epithelial integrity post-injury.

### Wound-induced polyploidization subverts the genotoxic stress response

**Fig. 7.**
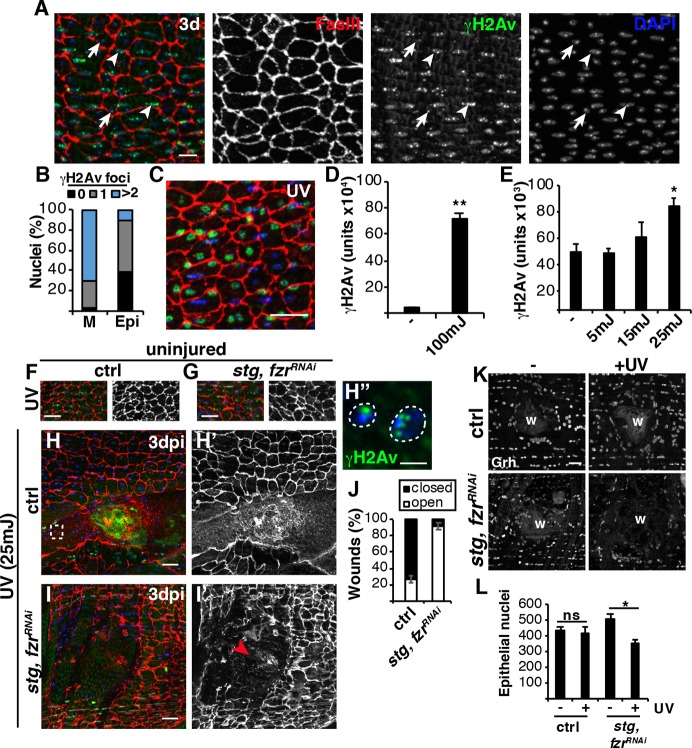
**Polyploidization enables wound repair in the presence of DNA damage.** (A) Immunofluorescent images of γH2Av staining (green) in abdomens of 3-day-old flies. Examples of muscle nuclei (arrowheads) and epithelial nuclei (arrows)*.* FasIII (red) and DAPI (blue). Scale bar: 10µm. (B) Quantification of γH2Av foci (*n*=200 nuclei). (C-E) UV irradiation enhances DNA damage in the epithelium. (C) Immunofluorescent image of γH2Av staining in abdomen exposed to 100 mJ of UV irradiation. Scale bar: 20 μm. (D) Quantification of γH2Av intensity in epithelial nuclei (*n*=50). (E) γH2Av intensity increases in a dose-dependent response to UV exposure (*n*=150). (F-H′,I,I′) Immunofluorescent images of fly abdomen exposed to 25 mJ of UV irradiation in either uninjured (F,G) or 3 dpi (H,I) animals. Scale bars: 20 µm. (H″) Boxed area in H with γH2Av (green) and DAPI (blue). Scale bar: 5 µm. (I′) Red arrowhead denotes open wound. (J) Quantification of wound healing at 3 dpi in control (*n*=22) and *stg, fzr^RNAi^* (*n*=26). (K) Switching to the mitotic cycle results in epithelial cell loss following UV treatment post-injury. Immunofluorescent image of epithelial nuclei (Grh) indicating strains ±UV irradiation at 3 dpi. Scale bar: 20 µm. Wound scab (W). (L) Quantification of the average number of epithelial nuclei per fly for control [−UV (*n*=4) and +UV (*n*=5)] and *stg, fzr^RNAi^* [ −UV (*n*=4) and +UV (*n*=5)]. *P<0.05; **P<0.01; ns, not significant. (Also see Table S7.)

The observed mitotic catastrophe could be due to the activation of mitotic cell cycle or a stress on the epithelial cells. Genotoxic stress, such as DNA damage, is one of the primary inducers of mitotic catastrophe (Vakifahmetoglu et al., 2008; Surova and Zhivotovsky, 2013). We stained the adult *Drosophila* abdominal tissue using phospho-histone 2Av (γH2Av), which binds to sites of DNA damage (Klattenhoff et al., 2007; Madigan et al., 2002). In *Drosophila,* as in other species, DNA damage is known to accumulate in muscle nuclei under conditions of physiological growth as well as with age (Bou Saada et al., 2017; Jeon et al., 2015). As expected, we observed robust γH2Av staining in lateral muscle nuclei in young (3-day-old) fruit flies with more than two γH2Av foci present in 71% of muscle nuclei (Fig. 7A,B). The *Drosophila* abdominal epithelial nuclei also stained with γH2Av and foci were apparent in 62% of the epithelial nuclei, with mostly a single γH2Av focus (Fig. 7A,B).

As we do not know the source of the epithelial DNA damage, we are not aware of any mechanism to reduce or eliminate it. However, epithelial DNA damage could be exacerbated by exposing the adult (3-day-old) fruit flies to UV irradiation (Fig. 7C). UV irradiation at 100 mJ resulted in robust induction of DNA damage, as evident by an ∼16-fold increase in nuclear epithelial γH2Av signal (Fig. 7C,D). The minimum, 25 mJ, UV irradiation dose significantly increased the γH2Av signal without causing damage to the adult epithelium (Fig. 7E-G). After UV irradiation, the WIP competent control flies were still able to efficiently heal, as 73% of wounds closed at 3 dpi with polyploid γH2Av^+^ epithelial nuclei visible (Fig. 7H,J). Re-epithelialization in the *stg, fzr^RNAi^* flies, however, was severely impaired as 92% of *stg, fzr^RNAi^* wounds remained open (Fig. 7I,J). In addition, we found that the switch to the mitotic cell cycle resulted in a significant loss of epithelial nuclei post-injury with UV treatment (Fig. 7K). There was no significant change in number of epithelial nuclei in the control, in which endoreplication occurs, but the *stg, fzr^RNAi^* mitotically induced epithelial cells were sensitive to UV-induced cell loss, as 152 epithelial nuclei were lost by 3 dpi after UV treatment (Fig. 7L).

**Fig. 8.**
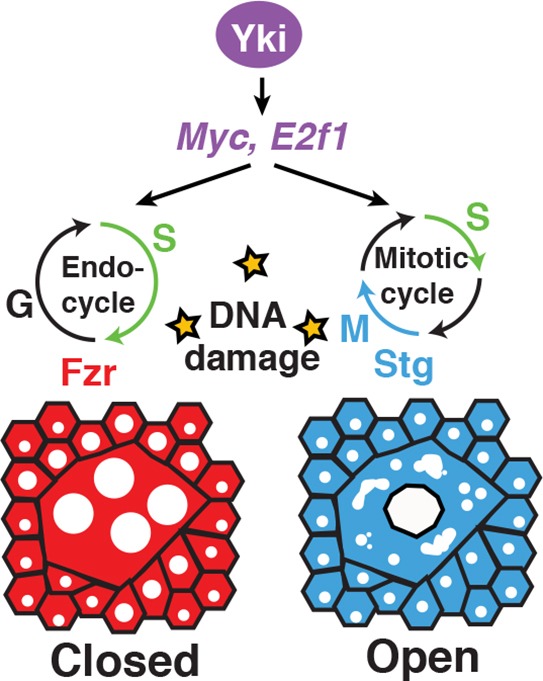
**WIP: an adaptive healing strategy.** Model illustrates the adaptive role of polyploid cell growth in enabling wound repair in presence of DNA damage. Yki-dependent induction of *Myc* and *E2f1* drives cells into the cell cycle (G/S) post-injury, but the expression of Fzr results in endoreplication and wound-induced polyploidization (WIP). The same Yki-dependent genes can also drive the mitotic cell cycle but, in the presence of DNA damage, they instead result in mitotic catastrophe and defects in wound closure.

Overall, our findings support a model in which polyploidy can be an adaptive wound repair strategy, particularly in cells that have accumulated DNA damage (Fig. 8). Genetically forcing the mitotic cell cycle through *stg, fzr^RNAi^*, results in mitotic errors that may be compounded by the previously incurred DNA damage of a tissue. Endoreplication is known to result in cells that are more resistant to genotoxic stress, thereby permitting more efficient wound repair (Hassel et al., 2014). In conclusion, we have found that *Myc* is a potent inducer of the endocycle even in post-mitotic epithelial cells, thereby determining their competence to enter the cell cycle. The expression state of the mitotic machinery of the cell then dictates whether the tissue repair program executed is either proliferation or polyploidization. In addition, we have found that polyploidization is an advantageous wound repair strategy when tissues have sustained prior DNA damage as it permits wound healing when mitosis would otherwise be compromised.

## DISCUSSION

### Proliferation versus polyploidization in tissue growth and repair

An unanswered question in tissue repair field is what limits cell proliferation? Why do some tissues retain the capacity to proliferate when injured, yet others fail to do so? Depending on the context (tissue and cell type) signaling pathways, such as the Hippo-Yki pathway, have been found to either promote cell proliferation or polyploidization, but the molecular mechanism regulating this choice of tissue growth has remained poorly understood. Here, we show that Yki induces a similar gene set (*Myc* and *E2f1*) for polyploid cell growth to that observed for cell proliferation. Myc and E2f1 are known to regulate the cell cycle at the G/S phase transition, but for cells to progress through mitosis, expression of mitotic regulatory genes is required. Here we find that Fzr, an E3 ligase that targets mitotic cyclins for proteolytic degradation, is expressed, while mitotic regulatory genes, including CycA and CycB, are repressed in the adult *Drosophila* epithelium. As a result, the Yki-dependent expression of *Myc* and *E2f1* induces an endocycle instead of mitosis to repair the adult fly epithelium. Interestingly, the conserved Hippo-Yap pathway has also been found to regulate both liver hepatocyte proliferation and polyploidization through mitotic arrest during tumorigenesis (Zhang et al., 2017). Therefore, the regulation of the mitotic machinery appears to be a conserved mechanism that may be used to determine whether tissues grow and repair by proliferation or polyploidization.

Some cell types appear to be more permissive than others to switching modes of tissue repair. In the mammalian heart, many studies have been performed to genetically or pharmacologically force cardiomyocytes to proliferate to improve heart regeneration (Zebrowski et al., 2016). However, majority of the adult cardiomyocytes are polyploid, which usually inhibits cell division. Only polyploid hepatocytes in the mouse liver and polyploid rectal papillae cells in *Drosophila* have been demonstrated to retain mitotic competence (Duncan et al., 2010; Fox et al., 2010). A recent study has shown that cardiomyocyte proliferation can be induced to improve heart regeneration by expressing of four cell cycle genes (*Cdk1*, *Cdk4*, *CycB* and *CycD1*) simultaneously (Mohamed et al., 2018). However, it was unclear whether the observed heart regeneration was due to polyploidy- or diploid-induced cardiomyocyte division, and the long-term effects on heart function caused by switching modes of repair.

In the *Drosophila* hindgut, diploid pyloric cells are induced into the endocycle in response to injury, and *fzr* knockdown was shown to be sufficient to switch to repair by cell proliferation instead of polyploid cell growth (Cohen et al., 2018). In adult fly epithelium, we found that the knockdown of *fzr* alone was not sufficient to switch to a mitotic cell cycle, but also required the ectopic expression of the mitotic activator *stg.* In addition, switching to a proliferative response in the fly epithelium significantly impaired wound healing, whereas the hindgut pylorus was not adversely affected by the switch and could still efficiently heal through cell proliferation instead of polyploidization. It was only upon additional oncogenic stress that a defect in tissue integrity in the hindgut was observed. This is also case in the mammalian liver, where polyploid hepatocytes have been shown to protect the liver from tumorigenesis (Zhang et al., 2018; Wilkinson et al., 2019). Therefore, the genetic factors necessary to switch modes of tissue repair are cell/ tissue dependent, with differences in both the short-term and long-term effects on tissue function.

### The relationship between DNA damage and polyploidy

The exposure to either physiological and/or damage-induced cytotoxic stress can result in cellular and genomic damage. Cytotoxic agents, including reactive oxygen species, are known to accumulate with age and injury resulting in DNA damage (Fortini et al., 2012). This accumulated DNA damage then poses a problem if cells attempt to proliferate by activating the DNA damage checkpoint and causing either apoptosis, cell cycle arrest, or mitotic errors (Surova and Zhivotovsky, 2013). However, polyploid cells have been found to have a higher resistance to genotoxic stress. Endoreplication was shown in *Drosophila* to result in chromatin silencing of the p53-responsive genes, allowing polyploid cells to incur DNA damage, but not die (Hassel et al., 2014; Qi and Calvi, 2016). Here we have shown that the adult *Drosophila* epithelium readily accumulates DNA damage, even at 3 days of age, yet the epithelial cells can circumvent this dilemma by inducing polyploid cell growth instead of cell proliferation upon injury.

It remains unclear why the adult epithelium readily accumulates DNA damage and whether WIP works through a similar mechanism to silence p53 targets. We tested for apoptosis activation in the mitotic-induced epithelial cells (*stg, fzr^RNAi^*), but could not detect any evidence of cell death using either the TUNEL or an active caspase 3 stain (data not shown). The mitotic errors in the epithelial cells may not activate cell death, as cell fusion was still observed (Fig. 6C). In the future, it will be interesting to address how polyploid cell generation by fusion contributes to the competence of cells to switch tissue repair modes.

### The role of CycE and Myc in cell cycle re-entry

Many tissues lack a resident stem cell population and to undergo efficient repair and regeneration the post-mitotic differentiated cells in the tissue must overcome the controls that restrain the cell cycle entry. A combination of growth factors and cell cycle regulators appears to be required (Pajalunga et al., 2008). In case of the *Drosophila*, Yki-dependent *CycE* expression was shown to be sufficient to promote cell cycle re-entry, resulting in cell proliferation following tissue damage in the eye imaginal disc (Meserve and Duronio, 2015). Here, we show that Yki-dependent *CycE* expression is also sufficient to trigger cell cycle re-entry following tissue injury, but results in endocycling instead of mitotic cell cycling. This was unexpected, as overexpression of *CycE* was shown to reduce salivary gland cell endoreplication in the *Drosophila* (Zielke et al., 2011). Overexpression of *CycE* blocks the relicensing of S-phase entry required for salivary gland cells to undergo successive endocycles and reach up to 1024C per nuclei. However, it is not a complete block as salivary gland cells still reached 64C with *CycE* overexpression (Zielke et al., 2011). Epithelial nuclei increase ploidy up to 32C, suggesting that CycE overexpression is not inhibitory for cells to undergo fewer than five endocycles. The overexpression of *CycE* without injury, however, was not sufficient to induce endoreplication. Conversely, *Myc*, another Yki-dependent target, efficiently overcame the cell cycle restraints to drive endoreplication even in the absence of tissue damage.

Myc regulates transcription of a large number of genes, which are required for cell growth, cell cycle and cell death (Bellosta and Gallant, 2010; Grifoni and Bellosta, 2015). In this study, we found that *Myc* is required and sufficient for post-mitotic epithelial cells to enter the endocycle and grow by becoming polyploid; however, ectopic *Myc* expression does not induce cell death, as has been observed in other systems. Myc has been shown to be activator of endoreplication in other *Drosophila* cell types, as well as in mammalian epidermal cells and megakaryocytes (Grifoni and Bellosta, 2015; Zanet et al., 2005; Avanzi et al., 2014). Although the Myc targets required to release the adult *Drosophila* epithelial cells from quiescence remain to be elucidated, Myc appears to be a potent inducer of cell cycle re-activation. Dormant adult muscle precursors in *Drosophila* larva also require a niche-induced Myc signal to re-enter the cell cycle and proliferate (Aradhya et al., 2015). In summary, activation of Yki by tissue injury induces a potent transcriptional gene set that is sufficient to cause cell cycle entry and is consistent with the previous finding that high levels of CycE and E2f1 are required to overcome cell cycle exit in terminally differentiated cell types (Buttitta and Edgar, 2007; Buttitta et al., 2010).

### Polyploidy: an adaptive repair response

In the past several years, an increasing number of examples of polyploidy have been observed not only in insects and plants, but also in vertebrate species, including zebrafish, mice and human tissue cell types (Gjelsvik et al., 2019). Polyploid cells are frequently generated in response to stress and/ or injury and are now recognized to offer an alternative tissue-growth strategy that can prevent acute organ failure (Lazzeri et al., 2019). Genotoxic stress is known to accumulate with age and has been observed in the mammalian cornea endothelium (Joyce et al., 2011), in which multinucleated polyploid cells are generated in response to damage or age-associated diseases (Ikebe et al., 1986, 1988; Losick et al., 2016). Acute injury to kidney also causes DNA damage and endoreplication in the tubule epithelial cells (Pressly and Park, 2017; Lazzeri et al., 2018). Therefore, it remains to be determined whether mitotic arrest allows polyploid cell growth to be the preferred tissue repair strategy to circumvent genotoxic stress in these mammalian tissues as well.

## MATERIALS AND METHODS

### Fly husbandry and strains

*Drosophila melanogaster* strains used in this study were reared on standard cornmeal agar yeast food at 25°C unless otherwise noted. The following *Drosophila* strains were obtained as indicated for use in this study. Bloomington (b), VDRC (v) and FlyORF (f) stocks numbers are indicated accordingly: R51F10-Gal4, referred to as epi-Gal4 (Losick et al., 2013); NP2108-Gal4 (Scherfer et al., 2013); *w^[1118]^* (b3605); PCNA-EmGFP (Deneke et al., 2016); *Myc-lacZ* (b12247); *ban-lacZ* (b10154); UAS-*yki^RNAi#1^* (v104523); UAS-*yki^RNA #2^* (b34067); UAS-*E2f1^RNAi^* (v108837); UAS-*CycE^RNAi^* (v110204); UAS-*Myc^RNAi #1^* (b36123); UAS-*Myc^RNAi #2^* (b51454); UAS-*banAS* (b60671); UAS-E2F-GFP (Duronio lab, UNC); UAS-CycE (b4781); UAS-Myc (b9674); CycB^CC01846^(Carnegie Protein Trap); *fzr-lacZ* (b12241); UAS-CycB (f001664); UAS-*fzr* RNAi (v25550); UAS-stg (b56562); and hid-GFP (b50750). The control used for all studies, unless otherwise noted, was the epi-Gal4/*w^[1118]^* strain. The following reporter strains were generated for this study: Myc-lacZ;; epi-Gal4*,* ban-lacZ, epi-Gal4 and NP2108-Gal4, PCNA-EmGFP, which were either crossed to the *w^[1118]^* as the control or UAS-transgene, as noted.

### PCR to verify *Drosophila* genotypes

*Drosophila* *yki* rescue strains generated for this study were verified by PCR. For further details, see the supplementary Materials and Methods.

### Fly wound assay, dissection and histology

Adult female flies 3-5 days old were punctured once on either side of the ventral midline between tergites A2 and A6 using a 0.10 mm stainless steel insect pin (F.S.T). At indicated times, abdomens were dissected in Grace's insect cell medium (Thermofisher) at room temperature under a light dissecting microscope. The internal organs were removed and the abdomens were filleted open by cutting along the dorsal midline with dissecting Vannas spring scissor (F.S.T). Filleted abdomens were pinned down on a Sylgard plate with four 0.10 mm insect pins and fixed while pinned open in 4% formaldehyde in 1× PBS for 30-60 min at room temperature. Antibodies and dilutions used in this study were rabbit anti-GFP (Thermofisher, A-11122, 1:2000), mouse anti-FasIII (DSHB, 7G10, 1:50), chicken anti-βgal (Abcam, ab9361 preabsorbed, 1:1000), rabbit anti-Yki (Pan lab, University of Texas Southwestern, Dallas, TX, USA), mouse anti-CycB (DSHB, F2F4, 1:50), mouse anti-CycA (DSHB, A12, 1:100), rat anti-Geminin (1:1000) (Quinn et al., 2001), rabbit anti-RFP (MBL, PM005, 1:1000), rabbit anti-H2AvD pSer137 (Rockland, 600-401-914, 1:1000), rabbit anti-Phospho Histone 3 (Cell Signaling, 9701, 1:1000) and rabbit anti-Grh (1:300) (Kim and McGinnis, 2011; Losick et al., 2016). Secondary antibodies from Thermofisher included donkey anti-rabbit 488 (A21206), goat anti-mouse 568 (A11031) and goat anti-chicken 488 (A11039) all used at 1:1000 dilution. Stained abdomens were mounted in vectashield on a glass coverslip, with the inner tissue facing out. Images were taken with 20×, 40× or 63× objectives, as indicated with Ziess ApoTome and *z*-stack projections compressed with Fiji/ImageJ software.

### EdU assay and quantification

Flies were fed 75 µl of 5 mM EdU yeast slurry 2 days prior to injury which continued until 2 dpi. EdU was detected according to manufactures instructions (Click-it EdU Imaging Kit, Thermofisher). The number of EdU^+^ nuclei was quantified from immunofluorescent images taken at 20× providing images of 666 µm×666 µm, which encompassed approximately half of the ventral abdomen.

### Ploidy quantification

*Drosophila* abdominal tissue was imaged using a Zeiss ApoTome 40× objective and processed with Fiji/ImageJ software to compile a SUM of *z*-stack projections. Images were rotated and cropped to a 333 µm×333 µm area. The control uninjured epithelial nuclei were used as an internal control for ploidy measurements by staining and imaging tissues under the same conditions and settings. This modification produced similar results to our previous published studies (Losick et al., 2013, 2016). Using Fiji, thresholded regions were drawn around each nucleus based on staining with the epithelial-specific nuclear marker Grh. ROI set regions were recorded and transferred to the corresponding DAPI SUM of the *z*-stack image. The DAPI intensity was measured within each outlined nuclear region. The average background was calculated and subtracted from the measured DAPI intensities. The ploidy was calculated by normalizing the DAPI intensity of the average value of the 2C uninjured epithelial nuclei for at least three abdomens per condition. The normalized ploidy values were binned into the indicated color-coded groups: 2C (0.6-2.9C), 4C (3.0-5.9C), 8C (6.0-12.9C), 16C (13.0-24.9C) and 32C (25-40). Nuclei that overlapped with other non-epithelial nuclei in the abdominal tissue were excluded from analysis.

### Detection of mitotic cell cycle

#### *CycB* fold change

*CycB* fold change was determined by measuring the integrated density of CycB staining in 3800 µm^2^ regions from 3 dpi samples compared with uninjured controls. In post-injury samples, regions quantified were adjacent to the wound site, but not overlapping with the wound scar, which has auto-fluorescence. The average CycB intensity from three regions were measured.

#### Epithelial nuclear number quantification

Epithelial nuclei were identified by their morphology and staining with epithelial specific transcription factor, Grainyhead (Grh) (Kim and McGinnis, 2011). Each sample was imaged at 20× and a 250 µm×250 µm section around the wound site, if injured, was quantified for the total number of epithelial nuclei.

#### PH3 quantification

Uninjured, 1 dpi and 2 dpi abdomens were stained using anti-PH3 and DAPI, and imaged using a Zeiss ApoTome at 40×. *Z*-stack images were processed with Fiji/ImageJ software as Max compression and the number of PH3^+^ epithelial nuclei were quantified for a 333 µm×333 µm area surrounding the wound site.

### Re-epithelialization assay

Wound closure was measured by scoring for the positive formation of a continuous epithelial sheet over the melanin scab using either staining for the septate junction marker FasIII or with epi-Gal4 expression of a membrane-bound UAS-mCD8-RFP. Only abdomens without processing perturbation were analyzed. Wounds were scored as closed (wound scab completely covered), partial (greater than 10 µm gaps in epithelium covering wound scab) or open (uncovered wound scab) at 3 dpi. Epithelial membrane thickness was measured by quantifying the epithelial mCD8.RFP intensity within a 1967 µm^2^ area covering the wound scab. Any wound with greater than 10 µm gaps in the FasIII-stained epithelium covering the wound scab was scored as a re-epithelialization defect for Fig. 5.

### DNA damage measurements and UV irradiation

The average number of γH2Av foci per nucleus was measured in 50 nuclei from four 3 day-old adult epi-Gal4 abdomens (*n*=200 nuclei). The average γH2Av staining intensity for lateral muscle or epithelial nuclei was measured in 25 nuclei per abdomen from four epi-Gal4 abdomens at indicated ages (*n*=100). Muscle and epithelial nuclei were distinguished by their position and morphology in abdominal tissue. Lateral muscle nuclei are elongated, whereas epithelial nuclei are smaller and round. Both are positioned in rows running across the ventral adult *Drosophila* abdomen.

UV irradiation was performed using a Hoefer crosslinker. Adult female flies, 3-5 days old, were anesthetized with FlyNap for less than 5 min and positioned with their ventral abdomen ∼6 cm from the UV bulb. Flies were injured 1 day after indicated UV dose and then dissected at 3 dpi.

### Statistical analysis

Experiments were performed at least twice with minimum of three biological replicates. The s.e.m. was calculated and significance measured using Student's *t*-test and Excel software: *P<0.05; **P<0.01; n.s., not significant (*P*>0.05).
